# Machine Learning Approaches for Quality Assessment of Protein Structures

**DOI:** 10.3390/biom10040626

**Published:** 2020-04-17

**Authors:** Jiarui Chen, Shirley W. I. Siu

**Affiliations:** Department of Computer and Information Science, Faculty of Science and Technology, University of Macau, Macau, China; mb85409@connect.um.edu.mo

**Keywords:** protein structure prediction, estimating model quality, model quality assessment, machine learning, deep learning, CASP, EMA, MQA, ML, DL

## Abstract

Protein structures play a very important role in biomedical research, especially in drug discovery and design, which require accurate protein structures in advance. However, experimental determinations of protein structure are prohibitively costly and time-consuming, and computational predictions of protein structures have not been perfected. Methods that assess the quality of protein models can help in selecting the most accurate candidates for further work. Driven by this demand, many structural bioinformatics laboratories have developed methods for estimating model accuracy (EMA). In recent years, EMA by machine learning (ML) have consistently ranked among the top-performing methods in the community-wide CASP challenge. Accordingly, we systematically review all the major ML-based EMA methods developed within the past ten years. The methods are grouped by their employed ML approach—support vector machine, artificial neural networks, ensemble learning, or Bayesian learning—and their significances are discussed from a methodology viewpoint. To orient the reader, we also briefly describe the background of EMA, including the CASP challenge and its evaluation metrics, and introduce the major ML/DL techniques. Overall, this review provides an introductory guide to modern research on protein quality assessment and directions for future research in this area.

## 1. Introduction

The three-dimensional structures of proteins are important biomolecular data in structure-based drug design [1,2]. Protein structures are usually determined by three techniques: X-ray crystallography, nuclear magnetic resonance (NMR) spectroscopy, and electron microscopy (EM). In X-ray crystallography, a protein structure is deduced from the unique diffraction patterns of the protein crystal. The molecular structure derived from X-ray experiments has always been considered as the most accurate structural model. However, as the purification and crystallization of proteins is very difficult and time-consuming, the number of solved protein structures remains much lower than the number of protein sequences. Meanwhile, NMR and EM require specialized equipment and facilities, which prevent their large-scale application. To overcome these problems, researchers have developed computational methods for protein-structure prediction. Popular methods include Modeller [3], SWISS-MODEL [4], Rosetta [5,6], I-TASSER [7], FALCON [8], Raptor/RaptorX [9,10], and IntFOLD [11] (see [12,13] for recent comprehensive reviews of the prediction theory and methods). Prediction functions are also available in some commercial software packages such as Internal Coordinate Mechanics, Molecular Operating Environment, and Schrödinger. Owing to their different algorithms and scoring strategies, these methods can predict very different structural models for the same protein sequence. For selecting the best predicted model, other means to evaluate the quality of a protein model are needed. Initially, a model selection function is included as a component in some structure prediction methods, but more and more independent methods have emerged in recent years. These methods are collectively called estimation of model accuracy (EMA) methods (formerly, model quality assessment methods). As their name suggests, these methods estimate how accurately the model fits the actual native structure, which is still unknown. A global-level EMA gives the average quality of a model, whereas a local-level EMA indicates the prediction quality of a segment of residues or a group of atoms. Due to the importance of model evaluation and ranking, Critical Assessment of Protein Structure (CASP) challenges started to assess EMA methods (QA category) since CASP7.

Machine learning (ML) and deep learning (DL) have proven their effectiveness in natural language processing, image processing, computer vision, speech recognition, and other computing domains. These successes have attracted the attention of researchers in bioinformatics and computational biology [2,14,15,16,17,18]. Thus far, ML and DL have been applied in protein classification and the predictions of protein structure and function, protein–ligand binding affinity, and protein–peptide/protein–DNA binding sites. Whereas traditional EMA methods are mainly based on energy, physicochemical, or statistical considerations [19], ML-based EMA methods combine multiple types of information. Recent methods can recognize the latent features such as protein contact pattern [20] and atom density map [21] from native structures. The superiority of ML-based EMA methods has been confirmed by their high rankings in CASP challenges.

**Figure 1:**
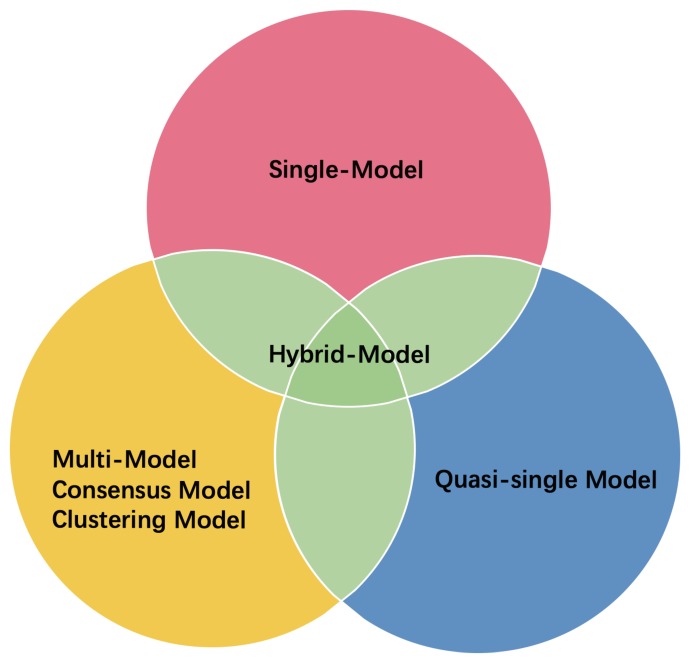
Four major approaches in the quality assessment of protein models: Single-model, multi-model, quasi-single model, and hybrid-model approaches. The hybrid-model methods combine selected models based on different methods.

ML-based EMA methods can be categorized into four major types (Figure 1): single-model, multi-model (also called consensus or clustering models), quasi-single, and hybrid methods. Methods for single models perform inherent feature extraction, with no reliance on external predictors. Their predictions are mainly based on the geometric and energetic analysis of a single-protein structural model. In contrast, multi-model methods cluster and extract the consensus information from a pool of protein structural models generated by multiple methods or from different templates [22,23,24]. Multi-method models assume that the correct structure is embedded in the recurring structural patterns of the model ensemble [25]. Therefore, the performance of a multi-model method depends on the quality and size of the model pool. A large model pool (possibly including tens of methods and tens to hundreds of models [26]) provides an accurate structure, but at high computational cost. Before CASP11, multi-model methods always outperformed single-model methods. In CASP11, single-model methods surpassed multi-model methods because of advancements in energy features and ML techniques [19,27,28,29,30]. However, multi-model methods achieved spuriously high performance in CASP13 compared with single-model methods; this is due to the significant improvements of protein structure prediction methods in recent years, leading to a high-quality model pool [31]. Meanwhile, quasi-single methods score a model by referencing a set of models generated within their internal pipeline, rather than by pooling externally generated models. In this sense, they differ from multi-model methods [22,32]. Finally, hybrid or combined approaches [33,34,35] combine the quality scores or patterns of different EMA algorithms (both single-model and multi-model) by weighting or ML algorithms. The final scores are more accurate than any of the single scores.

This review focuses on the ML techniques currently used in EMA methods. The remainder of the review is organized as follows. In Section 2, we briefly introduce the concepts and recent progress of ML, protein-structure prediction, the CASP challenge, and the popular features with data sources for training and evaluating ML-based EMA methods. After screening the citations, representativeness, reproducibility (available server or source code), and release time, we obtained 17 applications. Section 3 lists and compares these 17 applications in detail. Finally, we summarize the current developments and highlight the challenges and future directions of EMA research.

## 2. Background

### 2.1. Machine Learning and Deep Learning

ML is the technique by which computers learn from experience. The ML process resembles human learning activities. Given many examples or data, an ML algorithm formulates rules that map the data to the expected outcomes. Later, these rules are used for assessing unseen data and providing the probable correct answers. ML approaches can be broadly classified into four types [36]: supervised learning, unsupervised learning, semi-supervised learning, and reinforcement learning. The supervised learning approach derives knowledge from training data with labeled answers [37]. The learning process iteratively and automatically adjusts the inner parameters of the prediction model, with the goal of minimizing the prediction errors. Most of the EMA methods are based on supervised ML algorithms.

DL is a branch of machine learning. Conventional ML methods (like support vector machine and random forest) require manual feature design, selection, and extraction; but a DL method can learn the association between features and outputs automatically and extract complex descriptions from raw features internally, for example learning the hierarchical representation of data [38]. The usage of DL methods in structural bioinformatics raises the performance of predictive models to a new level [38,39,40]. The ML/DL algorithms and their use in EMA methods will be presented in Section 3.

### 2.2. Protein Structure Prediction

**Figure 2:**
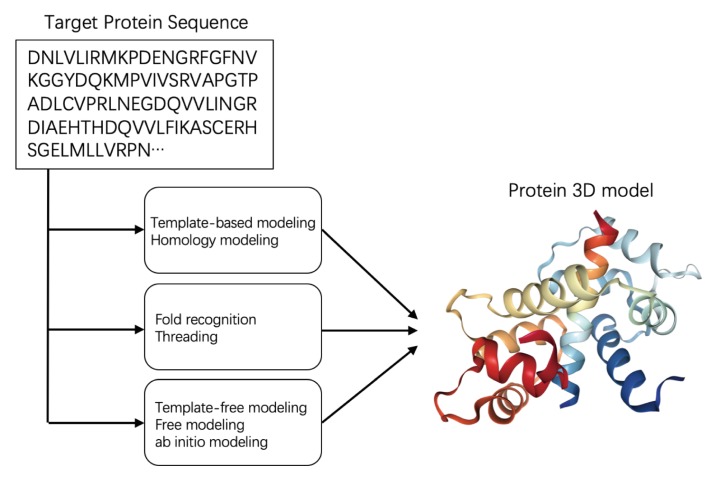
Three approaches of protein structure prediction.

Protein structure prediction, which attempts to predict the three-dimensional structure of a protein from its amino acid sequence [41,42], remains one of the most important and challenging problems in structural bioinformatics. Protein structures are predicted by three main approaches, as shown in Figure 2 [43].

The first approach, called template-based or homology modeling, allocates homologous proteins with known 3D structures as templates. Homology modeling is the most accurate of the three approaches when the quality of the templates is high, but if no homologous proteins with known 3D structures match the target, fold recognition (or threading) is preferred. Fold recognition assumes that natural proteins fold in similar ways. The target sequence is divided into fragments, and suitable fold structures for each fragment are searched from a fold library. Finally, the target structure is built by threading the sequence through the template folds. The third method, called template-free modeling or ab initio prediction, predicts the protein structures from scratch. After a conformational search of an initial peptide chain, this approach generates a large number of structure decoys, then ranks them by a scoring function that assesses their folding free energies. The best model is then selected as the decoy with minimum energy. As the folding prediction requires large computing power for modeling and searching, but has limited accuracy, this method is used only for predicting small proteins with up to 100 residues [44].

Protein structure prediction methods use protein properties such as secondary structure, relative solvent accessibility, backbone dihedrals, and contact maps inferred from the given amino acid sequence to build predictive models [39]. When homologous sequences of the target protein are available, multiple sequence alignment (MSA) of the sequences can be used for predicting these properties, and in turn using these properties to predict the protein structure. A key advancement in protein structure prediction is the exploitation of residue-residue contact prediction based on coevolutionary data from MSA [45] with the direct coupling analysis (DCA) techniques [46,47,48,49]. However, these co-evolution techniques are still not effective for those sequences that lack homologs [40]. The latest development on protein structure prediction involves direct extraction of sequential and pairwise features for inter-residue distance prediction in a global context [40], which is brought about by some DL-based methods, such as AlphaFold [50,51], MULTICOM [52], and RaptorX_Contact [40].

### 2.3. Critical Assessment of Structure Prediction

The CASP challenge, established in 1994 [53], is a community-wide contest that aims to benchmark the protein-structure prediction methods and stimulate advancement of the field. The challenge is designed for an accurate, comprehensive, and fair assessment of prediction methods. The way of assessing the methods has evolved over the years. In the last challenge, CASP13, eight categories of modeling aspects were independently assessed. These categories included high-accuracy and low-accuracy prediction of tertiary structure (i.e., template-based and free modeling, respectively), contact prediction, estimation of model accuracy (QA), quaternary assembly, model refinement, data-assisted prediction, and biological relevance. Since CASP7, CASP has been providing such a platform for evaluating EMA (QA) methods using the protein model structures submitted by the tertiary structure (TS) prediction servers [31,54,55]. These EMA methods have been assessed by a two-stage target-release procedure. In the first stage, sets of 20 structure models for each target are released for quality estimation. Selected from server models, these structure models span the whole range of model qualities rated by an in-house consensus method. In the second stage, a set of 150 models with similarly high quality is released for quality estimation. In both stages, the EMA methods must estimate the global quality of each structural model (global score) and the local quality of a model at the residue level (local score) [19,29,30]. The results of the first stage are only used to compare with the results of the second stage for the purpose of checking whether an EMA method is a single-model method [31]. The top-performing EMA methods in the CASP of a given year represent the state-of-the-art methods in protein prediction. Since CASP7, the EMA methods in CASP have improved on a yearly basis, driving EMA research to increasingly higher levels [19,20,27,28,29,30].

It is worth mentioning that the use of DL techniques has greatly improved the performances of the participating structure prediction methods. The overall accuracy of predicted models has improved dramatically in CASP13, especially for the more difficult targets that lack templates [13]. AlphaFold, the top-performing free modeling (FM) method in CASP13, includes one generative neural network for fragment generation and two deep residual convolutional neural networks for scoring, which together calculate inter-residue distances and evaluate structure geometry [50,51]. Another example is RaptorX_Contact, which has excellent performances in both CASP12 and CASP13, using a deep and fully convolutional residual neural network (ResNet) to predict protein contacts [40] (see [38] for a recent comprehensive review of DL-based structure prediction methods).

The significant progress made by these structure prediction methods has also imposed some challenges on EMA methods. This is because most EMA methods were developed based on previous CASP models, yet their performance evaluations were done on models generated by new structure prediction servers [31,54,55]. Although individual EMA methods showed progresses when compared to their previous versions, some of them performed worse than a pure consensus EMA method. This indicates that changing the quality of the generated models may affect the performance of those methods that implement consensus scoring [31].

To facilitate comparison of the state-of-the-art EMA methods, Table 1 presents seven EMA methods from six top-performing groups in CASP13 [20]. These EMA applications were selected for their high performances on global quality prediction (including top one loss and absolute accuracy estimation) and local quality prediction (including local accuracy estimation and inaccurately modeled regions prediction). Performance comparison of all EMA methods in CASP13 can be found at: http://predictioncenter.org/casp13/qa_diff_mqas.cgi.

### 2.4. Metrics

The accuracy of a predicted structural model is measured by its similarity to a corresponding experimental model; the higher the similarity, the higher is the accuracy and the better is the quality of the model. Structural comparisons are often quantified by the root mean squared deviation (RMSD), which is highly sensitive to large local deviations. Therefore, the RMSD score may not reflect the true accuracy of the model; moreover, it cannot properly rank very different or incomplete models (e.g., models with missing residues). To overcome the shortcomings of RMSD, researchers have developed other evaluation metrics, most notably the global distance test total score (GDT_TS), the template modeling (TM) score, the local-distance difference test (lDDT) score, the contact area difference (CAD), and SphereGrinder [60]. Ideally, the EMA methods will provide quality estimates that correlate to the computed evaluation metric scores (which are treated as the ground truth for this purpose).

1. GDT_TS [61,62]:
  The GDT is a rigid-body measure that identifies the largest subset of model residues that can be superimposed on the corresponding residues in the reference structure within a specific distance threshold [63]. The average GDT score (GDT_TS) within the specific distance thresholds provides a single measure of the overall model accuracy: [64]:
  (1) $$GDT\underline{\;\;}TS\left(M_{p},M_{r}\right)=\frac{\left(P_{1}+P_{2}+P_{4}+P_{8}\right)}{4},$$
  where $M_{p}$ is the predicted model, $M_{r}$ is the reference model, and $P_{1}$, $P_{2}$, $P_{4}$, and $P_{8}$ are the percentages of $C_{\alpha }$ atoms of $M_{p}$ that can be superposed on the $C_{\alpha }$ atoms of $M_{r}$ [65] within 1, 2, 4, and 8Å, respectively. The $GTD\underline{\;\;}TS$ score lies between zero (no superposition) and one (total superposition).
2. TM-score [66]:
  The TM-score of a structural model is based on the alignment coverage and the accuracy of the aligned residue pairs. This score employs a distance-dependent weighting scheme that favors the correctly predicted residues and penalizes the poorly aligned residues [67]. To eliminate the protein size dependency, the final score is normalized by the size of the protein. The TM-score lies between zero (no match) and one (perfect match) and is calculated as follows:
  (2) $$TM-score=max\left[\frac{1}{L_{ref}}\sum_{i}^{L_{aligned}}\frac{1}{1+{\left(\frac{d_{i}}{d_{0}\left(L_{ref}\right)}\right)}^{2}},\right]$$
  with:
  (3) $$d_{0}\left(L_{ref}\right)=1.24\sqrt[{3}]{L_{ref}-15}-1.8.$$
  Here, $L_{aligned}$ and $L_{ref}$ are the lengths of the aligned protein and native structure, respectively. $d_{0}(L_{ref}$) is a distance scale that normalizes $d_{i}$, the distance between a residue in the target protein and the corresponding residue in the aligned protein. The TM-score provides a more accurate quality estimate than GDT_TS on full-length proteins [66].
3. lDDT (LDDT in CASP) [65]:
  The lDDT score compares the environment of all atoms in a model to those in the reference structure, where the environment refers to the existence of certain types of atoms within a threshold. lDDT is advantaged by being superposition free. To compute the lDDT, the distances between all pairs of atoms lying within the predefined threshold are recorded for the reference structure. If the distances between each atom pair are similar in the model and the reference, this distance is considered to be preserved. The final lDDT score averages the fractions of the preserved distances over four predefined thresholds: 0.5Å, 1Å, 2Å, and 4Å [68]. The lDDT score is highly sensitive to local atomic interactions, but insensitive to domain movements.
4. CAD score [63]:
  The CAD score estimates the quality of a model by computing its interatomic-contact difference from the reference structure. The formulae are as follows:
  (4) $$CAD_{\left(i,j\right)}=\left|T_{\left(i,j\right)}-M_{\left(i,j\right)}\right|$$
  (5) $$CAD_{\left(i,j\right)}^{bounded}=min\left(CAD_{\left(i,j\right)},T_{\left(i,j\right)}\right)$$
  (6) $$CAD-score=1-\frac{\sum_{(i,j)\in G}CAD_{\left(i,j\right)}^{bounded}}{\sum_{(i,j)\in G}T_{\left(i,j\right)}}$$
  where *i* and *j* represent the residues in the predicted model and the reference protein structure, respectively, and *G* is the set of contacting residue pairs in the reference structure. $T_{\left(i,j\right)}$ and $M_{\left(i,j\right)}$ denote the contact areas in the reference structure and the predicted model, respectively. If a pair of contacting residues exists in the reference model, but not in the predicted model, that pair is excluded from set *G*. Similarly, if two residues contact in the predicted model, but are missing from the reference model, the contact area is regarded as zero. The CAD score ranges from zero (no similarity between the predicted and actual model structures) and one (perfect match of the predicted and actual structures).

The above scores determine the differences between the predicted (selected) structure and a reference structure. In some cases, the task of EMA methods is to predict one of the quality scores directly. These methods are evaluated by correlation and error (loss) between their predicted quality score with the ground-truth score. The resulting correlation coefficient represents the overall performance of the EMA method on the dataset, and the resulting error reflects the accuracy of the EMA method. The commonly used correlation coefficients are Pearson’s correlation coefficient (PCC) [69], Spearman’s rank correlation coefficient (Spearman’s ρ) [70,71], and Kendall’s rank correlation coefficient (Kendall’s τ) [72], and the most commonly used error scores are mean absolute error (MAE), mean squared error (MSE), and root mean squared error (RMSE) [21,73,74].

### 2.5. Features

ML-based EMA methods aim to evaluate the quality of a protein model. Their most important task is selecting a set of features representing the properties of a structure from different aspects. The features analyzed by ML-based EMA methods can be categorized into nine types. The feature categories and their applications in existing EMA methods are summarized in Table 2.

### 2.6. Data Sources

Training and validation are essential steps in ML-based EMA methods. A high-quality training dataset will improve the performance of the ML algorithm. Some commonly used data sources are listed in Table 3.

The CASP dataset consists of several sub-datasets (CASP1–CASP13). Samples from CASP7 to CASP13 are most commonly used for training and testing ML algorithms. Each protein target from the set is provided with hundreds of computer generated models (decoys). After pre-processing, these data are idealized for training and testing ML-based EMA methods.

The protein structure data in the PISCES and 3DRobot sources were selected from the Protein Data Bank (PDB) and organized by certain rules. The Continuous Automated Model Evaluation (CAMEO) project continuously evaluates prediction methods by different assessment criteria. As of 4 February 2020, CAMEO contained 50,187 structural models for model quality estimation [90]. CAMEO and CASP differ in two main respects: CAMEO contains fewer decoys per target than CASP, and its models have higher similarity than CASP models. The last dataset, the I-TASSER decoy set, is a non-redundant dataset containing 56 target proteins and 300–500 decoys per target [88]. In practice, several datasets should be combined to improve the training/test set of the ML algorithm. For example, the DeepQA method [74] combines the data from CASP8 to CASP10, 3DRobot, and PISCES as the training set and employs CASP11 data as the validation set.

### 2.7. K-Fold Cross-Validation

The accuracy of ML methods is commonly estimated by cross-validation (CV). A K-fold CV randomly partitions a dataset into K subsets. The model is trained on K−1 subsets, and the remaining subset is reserved for validating the model accuracy. Once all subsets have been validated, their accuracies are averaged to obtain the final performance measure. When the training data are insufficient or the CV is excessively time-consuming (as when training a DL model), the entire dataset can be split into two subsets (training and test) or three subsets (training, validation, and test) depending on whether model selection is required.

## 3. ML-Based EMA Methods

This section compares 17 EMA applications selected for their high popularity, ready availability, and performances in CASP. Most of these methods are based on artificial neural networks (NNs, CNNs, DBNs, and LSTM) and support vector machines (SVMs). Two methods are based on ensemble learning, and several methods use Bayesian learning (probability-based). Table 4 shows the details of these ML-based EMA methods.

### 3.1. Support Vector Machine

**Figure 3:**
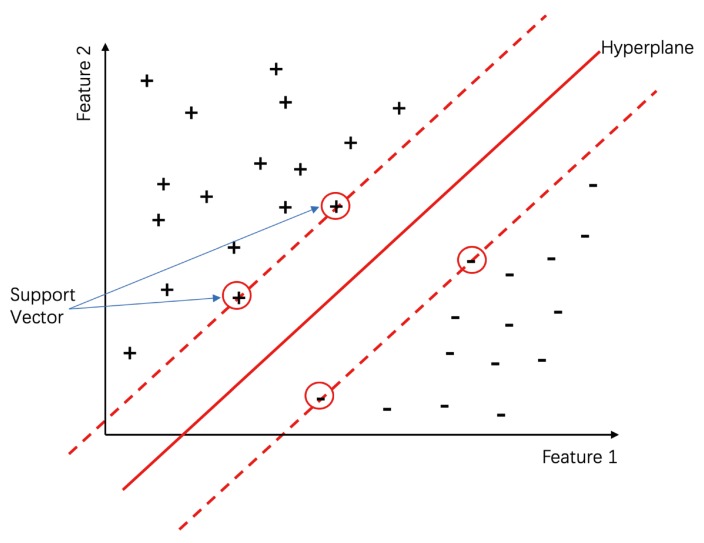
A simple illustration of SVM; + and - represent the sample labels.

SVM is among the most popular supervised learning techniques in classification and regression tasks [94]. In classification, SVM maps the original input feature space containing data from different classes, which are not linearly separable into a high-dimensional space, by a kernel function. Next, a hyperplane (see Figure 3) is sought by minimizing the risk of separating the data in each class. Three SVM-based EMA methods are presented below:

- ProQ2 & 3 [22,33]
  ProQ is a series of methods for EMA. ProQ2 selects the linear kernel function and a handful of structural and sequence-derived features. The former describes the local environment around each residue, whereas the latter predicts the secondary structure, surface exposure, conservation, and other relevant features [33]. ProQ3 inherits all the features of ProQ2, and adopts two new features based on Rosetta energy terms [22], namely the full-atom Rosetta energy terms and the coarse-grained centroid Rosetta energy terms. ProQ3 was trained on CASP9 and tested on CASP11 and CAMEO. ProQ3 outperforms ProQ2 in correlation and achieves the highest average GDT_TS score on both the CAMEO and CASP11 datasets [22].
- SVMQA [23]
  SVMQA inputs eight potential energy-based terms and 11 consistency-based terms (for assessing the consistency between the predicted and actual models) and predicts the TM-score and GDT_TS score [23]. This model was trained on CASP8 and CASP9 and validated on CASP10. In an experimental evaluation, SVMQA was the highest performing single-model MQA method at that time. The biggest innovation in this method is the incorporation of the random forest (RF) algorithm for feature importance estimation [23]. The features with higher importance are selected as the input parameters. Moreover, the quality score can be changed by varying the feature combinations. The TM-score (SVMQA_TM) is calculated from all 19 features, whereas the GTD_TS score (SVMQA_GTD) is determined from 15 features.

### 3.2. Neural Network

**Figure 4:**
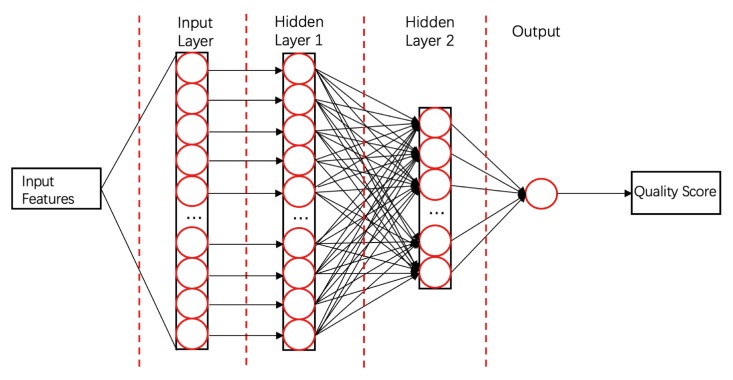
Diagram of the NN used by ProQ3D. Hidden Layers 1 and 2 contain 600 and 200 cells, respectively.

During training, the NN dynamically adjusts the weight of each neural cell based on the protein features and model quality score in the training set. Training ceases when the error rate of the NN falls below a certain level. At this time, the NN is considered as a well-trained model, and the pattern of its quality assessment is transformed into weight values for each cell. A trained NN can assess the quality of a new protein model or select the highest quality protein model from the model pool. Four methods adopt the NN in quality-assessment of a protein model:

- ProQ3D [58]
  ProQ3D includes all the features of ProQ3, but replaces the SVM model in ProQ3 with a multi-layer perceptron NN model containing two hidden layers. The first hidden layer contains 600 neural cells, and the second layer contains 200 neural cells and a rectified linear-unit activation function (as shown in Figure 4). Table A1 compares the performances of ProQ3D and its predecessors (ProQ, ProQ2, ProQ2D, and ProQ3) on the CASP11 data source. ProQ3D outperformed the other models in terms of Pearson correlation (0.90 for global quality, 0.77 for local quality), the area under the curve measure (AUC = 0.91), and GDT_TS score loss (0.006). As ProQ3D takes the same input features as ProQ3, the improvement is wholly and remarkably attributable to the improved learning model in ProQ3D. In the recent CASP13, the final version of ProQ3D outperformed ProQ3 in almost all measures [20]. It also performed as the second best single-model method in the “top 1 loss” analysis (ranking top model) of global quality assessment; this indicates that ProQ3D has great potential for global quality prediction.
- ModFOLD6 & 7 [34,57]
  ModFOLD is a series of EMA methods (the first version was pioneered by McGuffin [95] in 2008). ModFOLD6 and ModFOLD7 are the latest two generations, which were proposed for CASP12 and CASP13, respectively. Both methods achieved the best performance in the QA category of CASP. ModFOLD6 & 7 have similar working pipelines; different pure-single models and quasi-single models independently assess the features of a protein model and generate their own local quality scores. These local quality scores are considered as features and fed into an NN that derives the final predicted local score. Finally, the per-residue scores of the different methods are averaged to give the predicted global score. ModFOLD6 adopted ProQ2 [33], contact distance agreement (CDA) and secondary structure agreement (SSA) as pure-single methods and disorder B-factor agreement (DBA) [34,96], ModFOLD5 (MF5s) [97], and ModFOLDclustQ (MFcQs) [24] as quasi-single methods. ModFOLD6 was tested on CASP12 and part of the CAMEO set. Table A2 compares the performances of ModFOLD6 and other methods on CAMEO. The AUC score of ModFOLD6 (0.8748) far exceeded those of the other EMA methods (ProQ2, Verify3d, Dfire), and slightly surpasses that of ModFOLD4. This result demonstrates that a hybrid method has potential as a high-performing EMA method. In ModFOLD7, in order to improve the local quality prediction accuracy and the consistency of single model ranking and scoring, it adopts ten pure-single and quasi-single methods, including CDA, SSA, ProQ, ProQ2D, ProQ3D, VoroMQA, DBA, MF5s, MFcQs, and ResQ7 [98]. In CASP13, ModFOLD7 is one of the best methods for global quality assessment [31]. It provides two working versions of the method. ModFOLD7_rank is the best in ranking top models (assessed by the top one loss on GDT_TS and LDDT), and ModFOLD7_cor is good at reflecting observed accuracy scores or estimating the absolute error (based on the Z-score of GDT-TS differences and LDDT differences) [20].
- MULTICOM_cluster & MULTICOM_construct [20,31,52]
  Proposed by Hou et al., MULTICOM is a protein structure prediction method. Two sub-models, MULTICOM_cluster and MULTICOM_construct, had outstanding performances in the QA category of CASP13. They were the best methods in both the “top 1 loss” assessment (the top one losses on GDT_TS and LDDT were 5.2 and 3.9, respectively) and “absolute accuracy estimation” (based on Z-score of GDT-TS differences and LDDT differences) [31]. Similar to ModFOLD, MULTICOM uses a hybrid approach to assess the global quality of a protein model. Prediction results from 12 different QA methods (9 single-models, 3 multi-models) and 1 protein contact predictor (DNCON2 [99]) are taken as input features for 10 pretrained deep neural networks. Each of these DNNs generates one quality score for the given target model. For MULTICOM_construct, the final quality score is simply the mean of 10 quality scores predicted by DNNs. However, for MULTICOM_cluster, the combination of 13 primary prediction results and 10 DNN prediction results will be further put into another DNN for final quality score prediction. Their experiment showed that the residue-residue contact feature greatly improves the performance of the method, even though its impact varies depending on the accuracy of contact prediction. The success of MULTICOM has brought the residue-residue contact feature to the spotlight, such that it can consistently improve the performance of EMA methods adopting this or related features [20]. New advances in contact prediction based on DL and co-evolutionary analysis techniques may further improve EMA performance [40].

### 3.3. Convolutional Neural Networks

**Figure 5:**
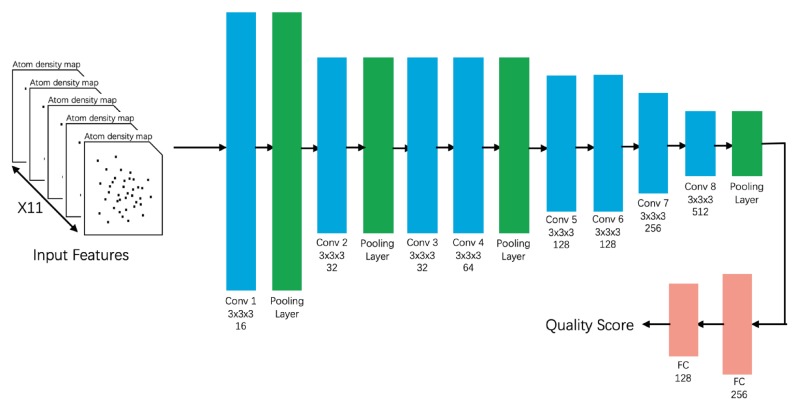
The convolutional neural network structure of 3DCNN.

Excellent CNN algorithms have emerged in recent years and have been widely exploited in image and speech recognition. Unlike traditional ML methods, a CNN learns a hierarchical representation directly from the raw data [91]. The convoluted data are input to an NN that performs the classification (see Figure 5). The direct use of raw data or low-level features, such as the protein residue sequence and protein-atom density maps, prevents information loss by feature selection and extraction. Furthermore, inputting raw data aligns with the end-to-end classification concept [91]. In CNN-based EMA methods, the 3D protein structure is usually regarded as an image, and the traditional manual feature extraction process is replaced with multiple convolutional layers. The different convolutional layers learn the extraction of different-level features from the 3D model during the training period. All features are then comprehensively considered and are combined to generate a final quality score for the protein model. Four exemplary CNN-based methods are introduced here:

- ProQ4 [21]
  ProQ4 inputs various protein structural features such as the dihedral angles φ and ψ, the protein secondary structure, the hydrogen bond energies, and statistical features of the sequence. The method has a multi-stream structure and trains each stream separately, which is feasible for transfer learning of the protein-structure quality assessment. ProQ4 was trained on CASP9 and CASP10 and tested on CASP11, CAMEO, and PISCES. On the CASP11 data source, ProQ4 delivered a poorer local performance than ProQ3D, but a significantly higher global performance. The local and global performances of ProQ4 and ProQ3D are given in Table A3 and Table A4, respectively. This method also proves the importance of the protein structure information in EMA. In addition, one of the main reasons for designing ProQ4 is to improve its target ranking ability. The result of CASP13 showed that ProQ4 successfully improved its target ranking over ProQ3D although its overall performance (GDT_TS, TM, CAD, and lDDT) was not better [20].
- 3DCNN_MQA [91]
  This state-of-the-art method inputs three-dimensional atom density maps of the predicted protein and analyzes 11 types of atoms. The success of this method proves the feasibility of inputting low-level raw data. During the training process, 3DCNN uniquely calculates the loss of the GDT_TS score rather than the GDT_TS score [91]. This method was trained on CASP7–CASP10 and validated on CASP11, CASP12, and CAMEO. The losses, Pearson’s correlations, Spearman’s correlations, and Kendall’s correlations of 3DCNN on CASP11 were 0.064, 0.535, 0.425, and 0.325 respectively in Stage 1 and 0.064, 0.421, 0.409, and 0.288 respectively in Stage 2 (Table A5). Unlike highly feature-engineered methods such as ProQ3D [58] and ProQ2D, this method uses simple atomic features, but is able to achieve moderate performance on CASP11.
- Ornate [92] & 3DCNN_MQA (Sato) [93]
  The original 3DCNN has two limitations, the resolution problem caused by different protein sizes and the orientation problem caused by different protein positions in 3D space. In order to solve these problems, Pages et al. developed Ornate [92] (oriented routed neural network with automatic typing), which is a single-model EMA method. Instead of using 3D density maps in the protein level, it breaks the density maps into residue level and aligns each map by the backbone topology, then these features are used as input data for a deep convolutional neural network. The local score is generated first, and the average of all local scores is taken as the final global score. Evaluation results on CASP11 and CASP12 showed that Ornate was competitive with most state-of-the-art single-model EMA methods. However, complex input features required by Ornate demand more processing time and computational resources during the learning process, which might be an obstacle for further improvement. Inspired by the original 3DCNN_MQA [91] and Ornate [92], Sato et al. proposed a new 3DCNN-based MQA method. There, the simplified atom categories and network topologies were used for predictive modeling so that the model could be more easily trained. The performances of 3DCNN_MQA (Sato) surpassed 3DCNN_MQA (Derevyanko) on the CASP11, CASP12, and 3DRobot datasets. The best performance of 3DCNN_MQA (Sato) was achieved in Stage 2 of CASP11; it outperformed all other state-of-the-art EMA methods on Spearman’s and Pearson’s correlation coefficients.

### 3.4. Deep Belief Network

The DBN [100,101,102,103] is essentially a stack of restricted Boltzmann machines (RBMs) that are consecutively trained to learn the latent factors from the data and make inferences from them. Unlike CNNs with convolutional layers, the DBN extracts a deep hierarchical representation of the data through the RBM network. Each RBM contains a layer of hidden units followed by a layer of visible units. The two layers are linked by undirected symmetrical connections, but the units within each layer are not connected (hence the term “restricted”). After training, the hidden units represent the latent factors of the data, providing a probabilistic explanation of the given input.

**Figure 6:**
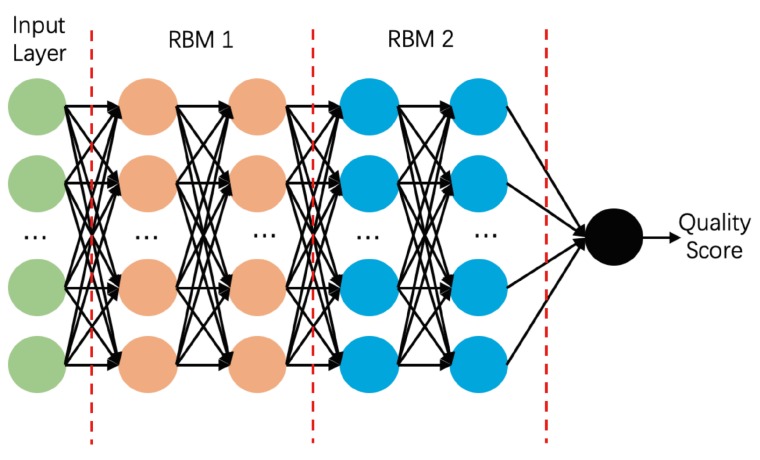
Structure of DeepQA. The neurones in each restricted Boltzmann machine (RBM) are independent and unconnected within the layers, but fully connected between the layers.

DeepQA [74] is a DBM-based model for quality evaluations of a predicted protein structure (see Figure 6). This single-model EMA method describes 16 structural, physicochemical, and energy properties for quality assessment. The features include the quality scores obtained by other top-performing EMA methods: the ProQ2 score [33], the Qprob score [35], and the ModelEvaluator score [73]. DeepQA contains two layers of RBMs for feature analysis and one layer of logistic regression nodes for the output target score (GDT_TS). The training sets of DeepQA are CASP8–10, 3DRobot, and PISCES. First, the network was coarsely trained by unsupervised learning; second, it was fine-tuned by the Broyden–Fletcher–Goldfarb–Shanno (BFGS) algorithm [104]. After the first training stage, the per-target correlation and loss of DeepQA on CASP11 were 0.64 and 0.09, respectively. This performance was comparable to that of ProQ2 (the top-performing single-model EMA method on CASP11). After the second training stage, the per-target average correlation and per-target average loss were improved to 0.42 and 0.06, respectively, outperforming the other methods (see Table A6 for the performance comparison on the CASP11 dataset [74]).

### 3.5. Long Short-Term Memory

**Figure 7:**
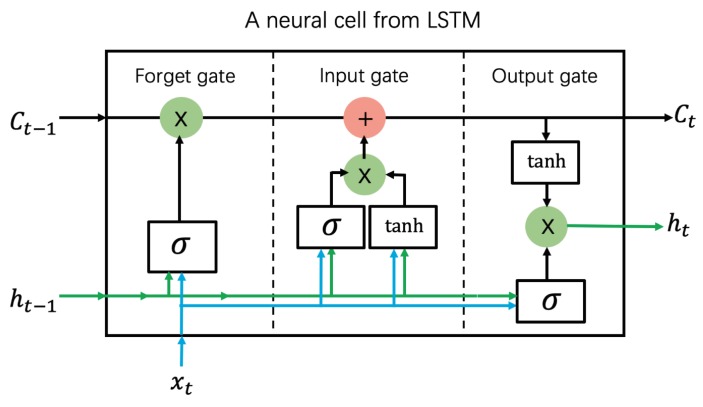
Simplified neural cell of LSTM, showing its three gates. $C_{t}$, $x_{t}$, and $h_{t}$ represent the cell state, input, and output in time step *t*, respectively.

LSTM is a special type of recurrent neural network (RNN) [105,106,107,108], originally designed to mitigate gradient explosion or disappearance on the RNN. As shown in Figure 7, a conventional LSTM neural cell is comprised of three basic units: an input gate, a forget gate, and an output gate. In this architecture, the hidden neurons of the LSTM remember the input data over a certain number of time steps. LSTM is especially competent at tasks involving sequence data, such as text translation and video processing.

Recently, Conover et al. [78] introduced a novel LSTM model called AngularQA for protein quality estimation. The core features of AngularQA are the angles between and within the protein residues. The Tau, Theta, Phi, and Delta angles in this method are weakly correlated to the GDT_TS score. Conover et al. also considered the amino acid type, secondary structure, protein properties (hydrophobicity, polarity, charge), and proximity counts of the residues [78]. In each time step, the features of one residue are input to the LSTM network for evaluation. Once the LSTM has processed the complete residue information of a protein model, it computes the GDT_TS score of that model. This method was trained on CASP9–CASP11 and 3Drobot and validated on CASP12. Because LSTM needs a continuous data flow, it cannot process protein models with missing residues and other discontinuities, which are thus excluded from the dataset.

In Stage 1 and Stage 2 of CASP12, the performance of AngularQA was not outstanding (see Table A7). In stage 1, the average per-target correlation and average per-target loss of AngularQA (0.545 and 0.116, respectively) were outperformed by ProQ3 (0.638 and 0.048, respectively) and DeepQA (0.654 and 0.078, respectively). The same trend could be observed in Stage 2. Despite its less than stellar results on CASP12, LSTM is a promising avenue in EMA research [78].

### 3.6. Ensemble Learning

Ensemble learning combines the predictions of multiple learners to improve the predictive performance [109]. All learners learn from the same dataset, or a dataset that has been modified by bootstrapping or weighting; meanwhile, the learning algorithms can be the same or different. The ensemble learners usually outperform the single learners, achieving higher generalization and lower variances.

The most widely used ensemble learning algorithm is RF, proposed by Breiman in 2001 [110]. This algorithm assembles hundreds or thousands of decision trees (DTs) for classification or regression tasks. During the prediction process, the input features are passed from the root to the end nodes of all DTs based on predefined splits, and the final RF is averaged over the outputs of all DTs [80]. The training process analyzes the feature importance values, thereby boosting the robustness of the learner in high-dimensional feature spaces or noisy data situations. One RF-based EMA method is RFMQA (2014), which predicts the TM-scores from statistical potential features (dDFIRE, Rwplus, and GOAP), the secondary protein structure, and the solvent accessibility information. In evaluations, RFMQA better discriminated the best protein-structural model than single-model and consensus methods, and the TM-score of its selected model was well correlated with that of the best model [80].

In 2016, Mirzaei et al. [84] proposed the MESHI-score, which also estimates the quality scores of protein decoys by EL methods. The MESHI-score is computed from 1000 predefined independent predictors, each of which inputs 60 physicochemical, energy, and meta-energy terms and generates a quality score (a GDT_TS score) for the given protein model. The final quality score is the weighted median of the 1000 scores [84]. The MESHI-score was trained on CASP8 and CASP9 and evaluated on CASP10. In an experimental evaluation, the MESHI-score better estimated the protein quality than the comparative model (SVM-e), which was trained on the same input features by a different learner.

### 3.7. Bayesian Learning

Most EMA methods predict the quality score of a protein structural model by Bayesian learning or probability-based approaches, which calculate the probability or probability density function (PDF). The PDF parameters are estimated from the training data, and the resulting PDFs provide the quality score of the protein structure. One Bayesian learning-based approach is Qprob [35], which takes 11 input features (three energy-based features and eight structural features) and computes the mean and standard deviation of the prediction errors of all targets for each feature type. Using these values, it then adjusts the predicted score of a new target using one feature and estimates the probability of that score. To minimize the average GDT-TS loss, each feature is assigned a weight by the expectation-maximization algorithm. Finally, the probability scores are combined to generate the final quality score of the protein model. Interestingly, although the prediction error distributions of most features appear to be non-Gaussian, the method achieves good performance. Qprob was both trained and tested on CASP9, PISCES, and CASP11. The experimental results verified Qprob as one of the best single-model EMA methods of its time. The method performed especially well on template-free protein structural models [35]. As the first attempt at quality-score estimation by error-based PDF, Qprob demonstrated the feasibility of probability-based methods in the quality assessment of protein models.

## 4. Summary and Future Perspectives

Motivated by the importance of protein structures, researchers have actively sought quality assessment methods for protein models over the past two decades. With modern advances in ML algorithms, ML methods have become the mainstream techniques for protein quality assessment, and their prediction quality has remarkably improved. After reviewing the major applications and breakthroughs of ML-based EMA methods, we made four observations:

First, most of the EMA methods are single-model methods. This trend is reflected in the number of single-model EMA methods in the CASP of each year, which increased from five in CASP10 to 22 in CASP12 and 33 in CASP13 [19,31].

Second, NN and SVM are the most popular techniques. The surging popularity of DL has increased the number of CNN-based EMA methods in the past three years [21,54,91]. These methods learn from only a few low-level input features, which promises to eliminate or reduce the effort of heavy feature engineering.

Third, a systematic and quantitative performance comparison of ML-based and non-ML-based methods is precluded because the benchmarks, EMA tasks, and training/evaluation data differ between the two method types. Nevertheless, the superior performance of ML-based methods over non-ML-based methods is evidenced by two facts: the popularity of ML-based approaches in EMA methods and the excellent performance of ML-based approaches in CASP. The former trend is reflected in the increasing number of ML-based EMA methods in recent CASP challenges. In the last CASP (CASP13), the 18 top-performing EMA methods proposed by six groups/laboratories included 12 NN-based methods, two SVM-based methods, three linear regression methods, and one knowledge-based potential method [20]. All of these methods except the last are related to ML. Moreover, ProQ2 was the most successful EMA method in the CASP11 challenge [30], whereas SVMQA and ProQ3 selected the best models from the model pool with excellent performance. These three methods are SVM-based EMA methods. In addition, the NN-based ModFOLD6 method reasonably predicted the global quality score in CASP12 [19,111]. These performances also highlight the excellent performance of ML in the quality assessment of the protein structure.

Fourth, the emergence of deep learning techniques has profoundly affected the performance of protein structure prediction methods. With the high quality protein models generated by DL-based prediction servers, the difficulty for EMA methods to differentiate these models accurately has increased. It is important to note that the pool of high quality models might lead to spuriously good performance in consensus methods as seen in the CASP13 assessment [31]. As most EMA methods are always trained on previous CASP models, this also poses the question of how the next generation EMA methods can meet the more stringent requirements of the ever-improved high quality models.

ML-based EMA methods are certainly meritorious, as on average, the best EMA methods select models that are better than those provided by the best server; however, so far, no single EMA method can always select the best model for a target [20]. This suggests that the best ML-based EMA methods are yet to come. Most of the ML algorithms are inputted with multiple features such as energy-based features, basic physicochemical features, and statistical features. Experimental results show that inputting different feature categories and different combination of features can change the performance of the algorithm [84,85]. Therefore, the features must be carefully selected. Finding the best feature combination is a future research direction. Although the RF algorithm is available for feature screening [23], it is not widely used for this purpose. On the other hand, because CNN-based EMA methods use the low-level (raw) features, they negate the need for feature screening. For example, the only input features of 3DCNN_MQA are 11 types of atom density map.

Meanwhile, the optimal use of ML in model accuracy evaluations is underdeveloped [20]. The number of new DL approaches increases each year, providing increasingly advanced ML approaches for EMA research. For example, AngularQA [78], which has been recently proposed for quality assessment of protein structures, is the first EMA method built with the LSTM architecture. Innovative ML approaches provide another avenue for improving current EMA methods. For example, ProQ4 [21] has a multi-stream network architecture and adopts an innovative transfer-learning approach. These constructs improve the global-score prediction and the selection from the model pool.

## Figures and Tables

**Table 1:** Top-performing estimating model accuracy (EMA) methods in CASP13 [20].

Name	Approach	Ref.	Advantage/Use Case	Available	S/C ^1^	Links
FaeNNz	NN	[20,56]	Top 1 accuracy estimate, absolute accuracy estimate	Y	C/S	https://swissmodel.expasy.org/qmean/
ModFOLD7	NN	[34,57]	Both global and local accuracy estimates	N	-	-
ProQ3D	NN	[31,58]	Top 1 accuracy estimate	Y	S	http://proq3.bioinfo.se/
ProQ4	CNN	[20,21]	Per target ranking	Y	C	https://github.com/ElofssonLab/ProQ4
SART	Linear regression	[31,57]	Local accuracy estimate and predict inaccurately modeled regions	N	-	-
VoroMQA	Statistical potential	[20,59]	Local accuracy estimate and predict inaccurately modeled regions, good for native oligomeric structures	Y	C/S	http://bioinformatics.ibt.lt/wtsam/voromqa
MULTICOM	NN	[31,52]	Top 1 accuracy estimate, absolute accuracy estimate	Y	C/S	http://sysbio.rnet.missouri.edu/multicom_toolbox/

^1^ “S” denotes server, and “C” denotes code.

**Table 2:** Categories of commonly used features in ML-based EMA methods.

Categories	Abbr.	Brief Description	Examples
Physicochemical properties	PC	Basic physical or chemical properties extracted directly from the protein structural model	Residue or atom contact information, atom density map, hydrophobicity, polarity, charge, dihedral angle, etc.
Surface exposure area	SE	Features calculated from the different types of a molecule’s surface area	**ProQ2**: Surface area [33] **DeepQA**: Exposed surface score [74] **Qprob**: Surface score of the exposed nonpolar residues [35]
Solvent accessibility	SA	Features based on the molecule’s surface area that is accessible to solvent	**DeepQA/Qprob**: SSpro4 [35,74,75] **ProQ2**: Solvent accessibility (calculated by NACCESS) [33,76,77]
Primary structure	PS	Protein sequence or features calculated from the sequence	**Wang SVM/AngularQA**: Residue sequence [78,79] **ProQ4**: Self information and partial entropy [21]
Secondary structure	SS	Secondary structure or features calculated from the secondary structure	**ProQ4/ModFOLD6**: Secondary structure from the DSSPdatabase [21,34] **DeepQA**: Secondary structure similarity score, secondary structure penalty score [74]
Evolutionary property	EI	Features based on the protein profile providing evolutionary information, collected from a family of similar protein sequences	**Wang SVM/ProQ2**: PSI-BLAST profile [33,79]
Energetic properties	ER	Features based on different energy terms	**DeepQA/RFMQA**: dDFIRE, RWplus [74,80,81,82]
Statistical potential	SP	Features involving statistical calculation or statistical potential	**DeepQA/RFMQA**: dDFIRE, GOAP, and RWplus [81,82,83] **SVM e**: Residue pair potentials [84]
Properties from other evaluation methods	FOM	Scores or features directly generated by other prediction methods	**DeepQA/RFMQA**: RWplus [74,80,82] **ProQ3**: ProQ2 [22] **ModFOLD6**: ModFOLD5 [34] **QAcon**: ModelEvaluator [85]

**Table 3:** Several commonly used data sources for training and testing EMA methods.

Data Sources		No. of Structures/Targets	URLs	Reference
CASP	CASP 7	- ^1^	http://predictioncenter.org/download_area/	[53]
	CASP 8			
	CASP 9			
	CASP 10			
	CASP 11			
	CASP 12			
	CASP 13			
PISCES		- ^1^	http://dunbrack.fccc.edu/PISCES.php	[86]
CAMEO		50,187/ - ^2^	https://www.cameo3d.org/	[87]
3DRobot		300 per target/200	https://zhanglab.ccmb.med.umich.edu/3DRobot/decoys/	[88]
I-TASSERDecoy	Set I	12,500–32,000 per target/56	https://zhanglab.ccmb.med.umich.edu/decoys/	[82,89]
	Set II	300–500 per target/56		
MESHI		36,682/308	http://wefold.nersc.gov/wordpress/CASP12/downloads/	[84]

^1^ The amount of data varies according to the demands of researchers. ^2^ As of 4 February 2020.

**Table 4:** Comparison of different EMA methods.

Name	Year	Dataset	Approach	Ref.	Input Property Categories ^1^									Available	S/C ^3^	Links
					PC	SE	SA	PS	SS	EI	ER	SP	FOM			
ProQ2	2012	CASP7-9	SVM	[33]	•	•	•	∘	•	•	∘	∘	∘	Y	S	http://duffman.it.liu.se/ProQ2/
DL-Pro (NN) ^2^	2014	CASP	NN	[64]	•	∘	∘	∘	∘	∘	∘	∘	∘	N	-	-
RFMQA	2014	CASP8-10	EL	[80]	∘	∘	•	∘	•	∘	•	∘	•	Y	C	http://lee.kias.re.kr/RFMQA/RFMQA_eval.tar.gz
Wang deep1 ^2^	2015	CASP11	NN	[79]	•	∘	•	•	•	•	∘	∘	•	N	-	-
Wang deep2 ^2^	2015	CASP11	NN	[79]	•	∘	•	•	•	•	∘	∘	•	N	-	-
Wang deep3 ^2^	2015	CASP11	NN	[79]	•	∘	•	•	•	•	∘	∘	•	N	-	-
Wang SVM ^2^	2015	CASP11	SVM	[79]	•	∘	•	•	•	•	∘	∘	•	N	-	-
QACon ^2^	2016	CASP9, 11	NN	[85]	•	•	•	∘	•	∘	•	•	•	N	-	-
ProQ3	2016	CASP9, 11, CAMEO	SVM	[22]	•	•	•	∘	•	•	•	∘	•	Y	S	http://proq3.bioinfo.se/
SVM-e	2016	CASP8-10, MESHI	SVM	[84]	•	∘	∘	∘	•	∘	•	•	•	N	-	-
MESHI-score	2016	CASP8-10, MESHI	EL	[84]	•	∘	∘	∘	•	∘	•	•	•	N	-	-
DeepQA	2016	CASP8-11, 3DRobot, PISCES	DBN	[74]	∘	•	•	∘	•	∘	•	•	•	Y	C	http://sysbio.rnet.missouri.edu/bdm_download/DeepQA_cactus/
ProQ3D	2017	CASP9-11, CAMEO	NN	[58]	•	•	•	∘	•	•	•	∘	•	Y	S	http://proq3.bioinfo.se/
SVMQA	2017	CASP8-12	SVM	[23]	∘	∘	•	∘	•	∘	•	∘	•	Y	C	http://lee.kias.re.kr/~protein/wiki/doku.php?id=start
ModFOLD6	2017	CASP12, CAMEO	NN	[34]	•	∘	∘	•	∘	∘	∘	∘	•	Y	S	http://www.reading.ac.uk/bioinf/ModFOLD/
Qprob	2017	CASP9, 11, PISCES	BL	[35]	•	•	•	∘	∘	∘	•	∘	•	Y	S	http://calla.rnet.missouri.edu/qprob/
3DCNN MQA	2018	CASP7-10, 11-12, CAMEO, 3DRobot	CNN	[91]	•	∘	∘	∘	∘	∘	∘	∘	∘	Y	C	http://github.com/lamoureux-lab/3DCNN_MQA
ProQ4	2018	CASP9-11, CAMEO, PISCES	CNN	[21]	∘	∘	∘	•	•	∘	•	•	∘	Y	C	https://github.com/ElofssonLab/ProQ4
ModFOLD7	2018	CASP10-13	NN	[57]	•	∘	∘	•	∘	∘	∘	∘	•	N	-	-
MULTICOM	2018	CASP8-13	NN	[52]	•	∘	∘	∘	∘	∘	∘	∘	•	Y	C/S	http://sysbio.rnet.missouri.edu/multicom_toolbox/
Ornate	2019	CASP11-12	CNN	[92]	•	∘	∘	∘	∘	∘	∘	∘	∘	Y	C	https://team.inria.fr/nano-d/software/Ornate/
AngularQA	2019	3DRobot, CASP9-12	LSTM	[78]	•	∘	∘	•	•	∘	∘	∘	∘	Y	C	http://github.com/caorenzhi/AngularQA/
3DCNN(Sato)	2019	3DRobot, CASP11-12	CNN	[93]	•	∘	∘	∘	∘	∘	∘	∘	∘	Y	C	https://github.com/ishidalab-titech/3DCNN_MQA

^1^ “•”/“∘” denotes that this property is adopted/not adopted by the EMA method. ^2^ Owing to their relatively low popularity and availability of servers or source codes, these models are not reviewed here. ^3^ “S” denotes server, and “C” denotes code.

## Appendix A. The Performances of ML-based EMA Methods

**Table A1:** Performances of the ProQ models in CASP11 [58].

Model	CC-Glob	CC-Target	CC-Loc	CC-Model	AUC	GDT TS Loss
ProQ	0.60	0.44	0.50	0.39	0.78	0.06
ProQ2	0.81	0.65	0.69	0.47	0.83	0.06
ProQ2D	0.85	0.68	0.72	0.49	0.89	0.05
ProQ3	0.85	0.65	0.73	0.51	0.89	0.06
**ProQ3D**	**0.90**	**0.71**	**0.77**	**0.54**	**0.91**	**0.06**

CC-glob and CC-target are the Pearson correlations of the global model quality, calculated over the whole dataset and averaged per target, respectively. CC-loc and CC-model are the equivalent Pearson correlations of the local model quality. AUC is the area under curve in local predictions. Residues closer than 3.8 Å from their positions in the target are considered correct. GDT_TS loss is the average global distance test total score (GDT_TS) difference between the selected model and the best possible model of that target. Bold font—top performance.

**Table A2:** Comparison of local scoring performances of EMA methods in CAMEO6 [34].

Model	AUC	StdErr	AUC 0–0.1	AUC 0–0.1 Rescaled
**ModFOLD6**	**0.8748**	0.00096	**0.0508**	**0.5081**
ModFOLD4	0.8638	0.00099	0.0467	0.4669
ProQ2	0.8374	0.00107	0.0428	0.4283
Verify3d	0.7020	0.00134	0.0208	0.2081
Dfire v1.1	0.6606	0.00138	0.0168	0.1675

Twenty-six weeks of data collected between 29 April and 21 October of 2016. AUC = area under the ROC curve. StdErr = standard error in AUC score. AUC 0–0.1 = area under the ROC curve with false positive rate ≤ 0.1. This table is sorted by AUC score. Bold font—top performance.

**Table A3:** Local-score performance comparison of ProQ4 and ProQ3D on CASP11 [21].

Method	R-Local	RMSE Local	local R-per Model
ProQ3D	**0.84**	**0.125**	**0.61**
**ProQ4**	0.77	0.147	0.56

R-local: Pearson correlation between all local predicted and true scores in the dataset; RMSE local: root mean squared error between the aforementioned predicted local scores and their true values; R-per model: average Pearson correlation between the predicted and true scores of each model in the dataset. Bold font—top performance.

**Table A4:** Global-score performance comparison of ProQ4 and ProQ3D on CASP11 [21].

Method	R-Global	RMSE Global	R-per Target	First Rank Loss
ProQ3D	0.90	**0.080**	0.82	0.040
**ProQ4**	**0.91**	0.085	**0.90**	**0.022**

R-global: correlation between all global predicted and true scores in the dataset; RMSE global: RMSE between the global predicted and true scores; R-per target: average correlation of global scores of the models for each protein; First Rank Loss: average difference between the true scores of the best model and the top-ranked model for each target. Bold font—top performance.

**Table A5:** Performances of 3DCNN_MQA and other state-of-the-art MQA methods on CASP11 Stage 1 and Stage 2 [91].

Method	Loss ^1^	Pearson	Spearman	Kendall
*Stage 1*			
ProQ3D	0.046	0.755	0.673	0.529
ProQ2D	0.064	0.729	0.604	0.468
**3DCNN_MQA**	**0.064**	**0.535**	**0.425**	**0.325**
VoroMQA	0.087	0.637	0.521	0.394
RWplus	0.122	0.512	0.402	0.303
*Stage 2*			
VoroMQA	0.063	0.457	0.449	0.321
**3DCNN_MQA**	**0.064**	**0.421**	**0.409**	**0.288**
ProQ3D	0.066	0.452	0.433	0.307
ProQ2D	0.072	0.437	0.422	0.299
RWplus	0.089	0.206	0.248	0.176

^1^ Loss = $max_{i}\left(GDT\underline{\;\;}TS_{i}\right)-GDT\underline{\;\;}TS_{argmin(s_{i})}$: difference between the GDT_TS of the best decoy and the GDT_TS of the decoy with the lowest predicted score *s* [91]. Bold font—top performance.

**Table A6:** Comparison between DeepQA and other top-performing single-model QA methods on CASP11 (Stage 1 and Stage 2) [74].

Method	Corr. on Stage 1	Loss on Stage 1	Corr. on Stage 2	Loss on Stage 2
**DeepQA**	0.64	0.09	0.42	0.06
ProQ2	0.64	0.09	0.37	0.06
Qprob	0.63	0.10	0.38	0.07
Wang_SVM	0.66	0.11	0.36	0.09
Wang_deep_2	0.63	0.12	0.31	0.09
Wang_deep_1	0.61	0.13	0.30	0.09
Wang_deep_3	0.63	0.12	0.30	0.09
RFMQA	0.54	0.12	0.29	0.08
ProQ3	0.65	0.07	0.38	0.06

Corr.: average per-target correlation, Pearson’s correlation between the real and predicted GDT_TS scores of all models; Loss: average per-target loss, defining the difference between the GDT_TS scores of the selected model and the best model in the model pool.

**Table A7:** Global score performances of AngularQA on CASP12 (Stage 1 and Stage 2) [78].

Method	Corr. on Stage 1	Loss on Stage 1	Corr. on Stage 2	Loss on Stage 2
**AngularQA**	0.545	0.116	0.393	0.128
ProQ3	0.638	0.048	0.616	0.068
DeepQA	0.654	0.078	0.578	0.100
Wang1	0.462	0.170	0.256	0.144
QMEAN	0.342	0.174	0.292	0.125

Corr.: average per-target correlation, Pearson’s correlation between the real and predicted GDT scores of all models; Loss: average per-target loss, defining the difference between the GDT scores of the selected model and the best model in the model pool.
